# Optimizing vaccination scheduling during influenza outbreaks: a SEIAR-based model for balancing routine and emergency vaccination demands

**DOI:** 10.3389/fpubh.2026.1807474

**Published:** 2026-05-07

**Authors:** Xiaochen Ma, Qiang Huang, Shanshan Chai, Boteng Yin

**Affiliations:** 1 School of Economics and Management, Dongguan University of Technology, Dongguan, Guangdong, China; 2 Nanshan District Center for Disease Control and Prevention, Shenzhen, Guangdong, China; 3 College of Management, Shenzhen University, Shenzhen, China

**Keywords:** influenza control, resource optimization, SEIAR model, service capacity, vaccination scheduling

## Abstract

**Background:**

Efficient scheduling of vaccinations, particularly during influenza outbreaks, poses significant challenges due to limited service capacity and the need to balance routine immunization with emergency vaccination demands.

**Methods:**

We developed a mathematical optimization model integrating an SEIAR influenza transmission framework to determine optimal service capacity allocation. The model prioritizes routine Category I vaccinations while maximizing emergency influenza vaccination capacity. Model validation employed vaccination data from Nanshan District CDC, Shenzhen (*n* = 4,193 children, 11,528 vaccine doses) and an influenza outbreak at a local secondary school.

**Results:**

The optimized model reduced peak infection counts by 80.6% (from 360 to 70 individuals) and significantly decreased daily vaccination variance (from 38.25 to 3.17, 91.7% reduction), while maintaining 100% coverage for routine vaccinations. The optimal service capacity threshold was determined at 49 individuals per day.

**Conclusion:**

The proposed balanced vaccination scheduling model offers a practical solution for optimizing vaccination resource allocation during influenza outbreaks. By incorporating vaccination compliance and dynamically adjusting service capacities, the model provides a robust framework for improving vaccine management and epidemic control. This model can be applied in disease prevention and control centers to ensure efficient utilization of vaccination resources, particularly in outbreak scenarios.

## 1 Introduction

Since the twentieth century, outbreaks of infectious diseases have occurred with increasing frequency and geographic reach, accompanied by a marked escalation in their societal and public health impact. Notable examples include the A/H1N1 influenza, Ebola virus disease, Severe Acute Respiratory Syndrome (SARS), and the COVID-19 pandemic. During such outbreaks, rapid deployment of emergency vaccination campaigns has proven to be one of the most critical interventions for curbing transmission and reducing morbidity and mortality (1). Influenza outbreaks, in particular, often evolve within a short time window, requiring vaccination sites and public health agencies to mobilize service capacity quickly in order to contain further spread. However, emergency vaccination campaigns do not take place in a vacuum. In routine practice, vaccination clinics must simultaneously maintain essential immunization services for children, and any disruption to these services may result in delayed or missed doses with long-term consequences for population health. Thus, during an influenza outbreak, the central operational challenge is not only how to expand emergency vaccination, but also how to do so without undermining routine immunization delivery.

Routine childhood immunization is equally critical to public health. Since 1974, immunization programmes are estimated to have averted 154 million deaths worldwide, including 146 million among children younger than five years, of whom 101 million were infants under one year of age (2). In China, the national immunization program classifies vaccines into two categories. Category I vaccines (Expanded Programme on Immunization, EPI) are government-funded and mandatory for children, covering essential antigens including diphtheria, tetanus, pertussis, and polio, administered primarily to children aged 0–14 years (3). Category II vaccines, such as influenza vaccines, are voluntary and privately funded (4). Currently, the implementation of large-scale immunization programmes in China poses substantial operational challenges owing to the country's large population and the heterogeneity of vaccination requirements. Variations in dose numbers, inter-dose intervals (5) and age eligibility (6) substantially increase scheduling complexity, complicating appointment allocation and resource planning at local Centers for Disease Control and Prevention (CDC). In addition, during epidemic outbreaks, demand for influenza vaccination can surge within short time frames, frequently exceeding routine service capacity. This creates an inherent tension between sustaining routine immunization coverage and responding to acute outbreak-driven demand. Current vaccination scheduling practices in Chinese CDCs rely largely on fixed-cycle planning and manual adjustments, approaches that are poorly equipped to accommodate heterogeneous constraints and sudden demand shocks. As a result, ensuring high routine vaccination coverage while flexibly responding to emergency immunization needs requires more systematic and optimized scheduling strategies.

Recent years have witnessed growing academic interest in the optimisation of vaccination scheduling. Existing studies have developed a range of quantitative frameworks to support large-scale immunization programmes. For example, Zhang et al. (7) proposed a mass vaccination appointment scheduling model that jointly determines vaccination site locations, appointment acceptance, allocation and timetables by integrating population distribution and time-window constraints. Under conditions of uncertain vaccine supply, Karakaya and Balcik (8) developed a stochastic optimisation framework for national vaccination calendars that explicitly accounts for multi-dose regimens, heterogeneous dosing schedules and uncertainty in vaccine delivery, and demonstrated its applicability using COVID-19 vaccination data from Norway. Similarly, Fabbri et al. (9) introduced a mixed-integer linear programming-based decision support system for pandemic vaccination campaigns, substantially reducing planning time compared with manual scheduling approaches. More recently, Luo and Shechter (10) studied the mass vaccination scheduling problem that trades off infections, throughput, and overtime at vaccination centers, providing a queueing-based analytical framework for capacity-aware appointment scheduling during epidemics. Despite these advances, most existing models consider vaccination scheduling for a single vaccine type in isolation and overlook the coordinated management of routine immunization and emergency vaccination. In particular, few models incorporate the heterogeneous constraints that characterize pediatric immunization programmes, including age-dependent eligibility windows, multi-dose inter-dose interval requirements, and the regulatory classification of vaccines into mandatory (Category I) and voluntary (Category II) types as practiced in China (3).

In addition, another critical aspect of real-world vaccination programsrecipient compliance (i.e., the extent to which caregivers bring their children in for vaccinations on the scheduled dates) rarely addressed in the optimization literature. Machado-Alba et al. (5) reported high coverage and timeliness of childhood vaccination in a Colombian cohort, documenting that delayed or missed doses remain a persistent challenge, particularly for multi-dose vaccine series. Md Suhaimi et al. (1) examined the influence of maternal risk perception and vaccination knowledge on childhood vaccination intentions, providing empirical evidence that compliance rates are context-dependent and cannot be assumed to be uniform. At the global level, the Global Burden of Disease Study 2023 systematic analysis confirmed that childhood vaccination coverage has not returned to pre-pandemic levels, with disruptions most pronounced in the 0–14 age group, where coverage gains slowed significantly between 2010 and 2019 and declined sharply after 2020 (2). These findings underscore the urgency of developing scheduling models that explicitly account for imperfect compliance. However, in existing studies, recipient compliance is typically assumed to be perfect or implicitly ignored, limiting the ability of current approaches to capture operational uncertainty and reducing their applicability in real-world vaccination settings. In particular, the interaction between emergency influenza vaccination and mandatory childhood immunization programs including vaccination intervals, age-appropriate vaccination windows, and catch-up vaccination requirements has not yet been modeled within existing SEIAR-based frameworks.

Another area of research focuses on vaccine allocation, equity, and distribution logistics under resource constraints. Guo et al. (11) integrated vaccine demand allocation with delivery logistics, vaccination site location and population distribution to reduce travel, distribution and service costs while maximizing overall immunization coverage. Wen et al. (12) employed mixed-integer optimisation frameworks to incorporate population heterogeneity, accounting for vaccination willingness, prioritization policies and target-group differences, thereby improving the rationality of allocation decisions. From an equity perspective, Chen et al. (13) demonstrated that strategically prioritizing vaccination in vulnerable communities can simultaneously enhance social welfare and fairness, even in the presence of elevated vaccine hesitancy. Related studies have further explored the joint effects of hesitancy and capacity constraints on equitable vaccine distribution during pandemic responses (14, 15). In terms of distribution logistics, Tang et al. (16) investigated mobile vaccination routing and scheduling with capacity selection, formulating a capacitated vehicle routing problem to optimize community assignments and service sequences under resource constraints in Dalian, China. Despite these contributions, the existing literature has largely concentrated on vaccine allocation under acute pandemic conditions at national or regional scales. Far less attention has been paid to the coordination of vaccination resources in settings where routine immunization programmes must operate concurrently with emergency vaccination demands. Moreover, most models assume fixed or effectively unlimited service capacity at vaccination sites, overlooking the need for dynamic capacity adjustment in response to rapidly evolving epidemic conditions.

More recently, epidemiological transmission models have been increasingly integrated with optimisation frameworks to examine vaccination strategies and epidemic control. Hosseini-Motlagh et al. (17) developed control-oriented modeling approaches to inform intervention design during the COVID-19 pandemic. Cala ore et al. (18) investigated the dynamic planning of multi-dose vaccination campaigns under uncertain vaccine supply, highlighting the critical role of vaccination timing in reducing peak infection levels and cumulative case numbers. Hou and Bidkhori (19) proposed a multi-feature SEIR model that captures heterogeneity in health conditions and social activity levels among populations, improving predictive accuracy for epidemic analysis and vaccine prioritization. In the specific context of influenza, the SEIAR (Susceptible-Exposed-Infectious-Asymptomatic-Recovered) compartmental framework has been widely adopted to capture incubation period characteristics and the ability of asymptomatic individuals to transmit the virus. Chen et al. (20) employed a SEIAR model to simulate seasonal influenza outbreaks in schools in Changsha, China, and quantified the effectiveness of isolation, antiviral prophylaxis, and pre-outbreak vaccination. Kamrujjaman and Mohammad (21) modeled influenza transmission dynamics across Mexico, Italy, and South Africa using data from 2021 to 2023, performing sensitivity analysis to reveal the effectiveness of various prevention strategies including vaccination timing. Despite the growing sophistication of coupled epidemiological-optimization approaches, several limitations remain when viewed from the perspective of childhood vaccination scheduling during influenza outbreaks. Most existing analyses assume sufficient vaccine availability and focus on optimizing strategies at the population level, rather than on the operational scheduling problem of how many individuals can actually be served each day at a specific facility. Limited attention has been paid to resource allocation when vaccination site capacity is explicitly constrained.

In summary, despite substantial progress in vaccination scheduling optimisation, several critical limitations remain. Existing studies commonly address routine immunization and emergency vaccination as independent problems, overlooking the operational interdependence between the two. In addition, appointment adherence is typically assumed to be perfect or treated in a highly simplified manner, despite the fact that missed appointments by children and their guardians introduce significant uncertainty into scheduling feasibility and resource utilization. Moreover, vaccination scheduling models are rarely coupled with infectious disease transmission dynamics, limiting their ability to inform capacity allocation under epidemic conditions. It should be noted that several existing studies have modeled multiple vaccine types or multiple influenza subtypes, and the rationale for doing so deserves clarification. In the area of vaccine allocation, studies such as Guo et al. (11) and Wen et al. (12) incorporated heterogeneous vaccine types to reflect differences in target pathogens, dosing regimens, and eligible age groups. In influenza transmission modeling, multi-strain frameworks have been developed to capture the co-circulation of subtypes (e.g., H1N1, H3N2, and B lineages) that characterize typical influenza seasons (22). Such models are valuable for studying cross-immunity, strain replacement, and polyvalent vaccination strategies at the population level. However, this study addresses a different issue: the operational scheduling of daily vaccination capacity at a single vaccination center during a localized outbreak. Furthermore, the scheduling optimization framework is structurally independent of the number of subtypes and operates in the same manner regardless of whether the vaccine targets a single subtype or multiple subtypes.

Therefore, addressing these research gaps, this paper proposes a balanced scheduling optimization model to resolve the following core issues: (1) Determining the minimum daily vaccination capacity that vaccination sites should maintain while ensuring no child misses a Category I vaccine; (2) Dynamically allocating limited service resources between Category I vaccines and influenza vaccines during infectious disease outbreaks like influenza to maximize epidemic control effectiveness. This study uses transmission data from a community vaccination site in Nanshan District, Shenzhen, China, and an influenza outbreak at a Shenzhen secondary school as case studies. By integrating the SEIAR infectious disease dynamics model, it validates the effectiveness and practicality of the proposed model.

The innovations of this paper lie in:

(1) We develop a vaccination scheduling framework explicitly incorporating time-sensitive urgency and realistic appointment non-adherence, reducing missed vaccination risk through dynamic re-scheduling mechanisms.
(2) We couple the SEIAR transmission model with Category I vaccination scheduling to ensure routine coverage while maximizing influenza prevention capacity during outbreaks.
(3) By employing bisection search, we reduce computational complexity from *O*(*n*) to *O*(*log*2*n*), enabling real-time scheduling decisions for large vaccination programs.

## 2 Materials and methods

This study develops an integrated mathematical optimisation framework coupled with an influenza transmission model (SEIAR) to conduct numerical experiments and case-based analyses, with the aim of deriving a balanced vaccination scheduling scheme applicable to disease prevention and control centers and vaccination facilities. The proposed framework serves two primary functions. First, it generates balanced schedules for children requiring Category I vaccines, including those eligible for routine immunization as well as those requiring catch-up doses. Second, it coordinates the allocation of service capacity between Category I vaccines and influenza vaccination during outbreak periods, ensuring effective epidemic response without compromising routine immunization coverage. The practical applicability of the framework is evaluated through case studies based on two operational scenarios from the childhood immunization programme of a CDC in Shenzhen, China, encompassing both routine vaccination and influenza outbreak response.

**Figure 1:**
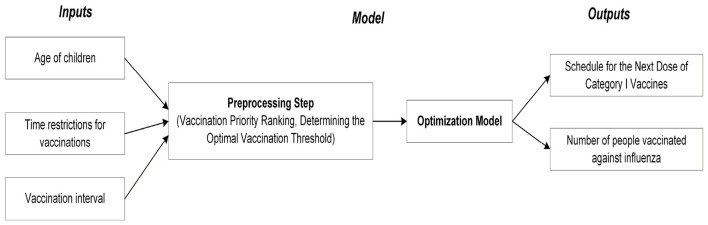
Schematic diagram of the approach.

The logic of this model is illustrated in Figure 1. Model inputs include the candidate vaccination interval under study, along with children's ages and vaccination time constraints. The preprocessing step prioritizes vaccinations based on children's ages and vaccination time constraints, then employs a binary search to identify the optimal dose limit. The objective function balances two goals: (1) determining the timing for the next Category I vaccine dose, and (2) maximizing influenza vaccination capacity while ensuring no missed Category I vaccinations, thereby optimizing the allocation of resources for both vaccination types.

### 2.1 Model parameters and notation

To better illustrate the problem and establish the model, reasonable assumptions have been made regarding relevant scenarios based on actual research findings, as follows:

(1) Each child receives at most one vaccine dose per day to minimize the risk of administration errors and to prioritize vaccine safety over scheduling convenience. Intervals between different vaccines comply with the 2016 Immunization Work Specifications (23).
(2) Population size is assumed to remain constant, with migration flows neglected.
(3) The population involved in influenza transmission is assumed to be homogeneous, with uniform contact rates and susceptibility, and behavioral changes affecting individual susceptibility are not explicitly modeled.
(4) For modeling simplicity, vaccination is assumed to provide complete protection once administered, such that vaccinated individuals are removed from the susceptible population.
(5) The influenza outbreak is assumed to be driven by a single dominant subtype, consistent with the case study data (24) in which the school outbreak was attributed to influenza B/Yamagata. This is a reasonable approximation for localized school outbreaks that are typically dominated by a single subtype (20).

The assumption of complete vaccine protection is a simplifying assumption introduced for mechanism identification. The primary purpose of the SEIAR component in this study is to evaluate how reserved vaccination capacity may alter outbreak trends under service-capacity constraints, rather than to estimate the exact field effectiveness of influenza vaccines. Meanwhile, this simplification does not affect the core scheduling structure of the model, because the problem addressed in this paper is the allocation of limited daily service capacity between Category I vaccination and influenza vaccination.

Table 1 summarizes the notation and definitions of all the parameters, sets, and variables used in the model. In particular, during the scheduling process for Category I vaccines, if the planned scheduling period differs from the actual period by T days, children who did not attend the vaccination center as scheduled will retain high priority and be re-scheduled in the next scheduling cycle (T days later). Additionally, when pre-scheduling all children's data, a daily vaccination capacity limit is set. The number of children scheduled per day does not exceed this limit. Based on priority, children requiring more urgent vaccination are selected for the next dose scheduling. When identifying the optimal daily capacity threshold, the model evaluates whether any child would miss a required dose under the corresponding schedule. In addition, the feasible age window for administering the next vaccine dose is jointly constrained by the vaccine-specific eligibility limits and the timing of previously administered doses, together with their required inter-dose intervals (see Appendix).

**Table 1:** The major parameters and notations.

Notation	Description
Parameters	
*T*	Single scheduling cycle.
*DT*	Rolling scheduling cycle.
*CT*	Inspection cycle.
*total*_*t*	Total scheduled time.
*I*	Set of all children.
*J*	Set of all vaccines, *J* = [1, ......*n*], *n* = 28.
*i*	The *i*-th child, where *i* ∈ *I*.
*j*	The *j*-th dose of the vaccine, where *j* ∈ *J*, excluding the 1st and 4th doses.
*L*_*j*_	The earliest age for receiving the *j*-th dose of the vaccine.
*O*_*j*_	The latest age for receiving the *j*-th dose of the vaccine.
*S*_*jk*_	The interval between the *j*-th dose and the previously administered *k*-th dose of the vaccine.
*a*_*it*_	On day *t*, the age of the *i*-th child.
*f*_*ijt*_	On day *t*, the age of the *i*-th child receiving the *j*-th vaccine dose.
*x*_*ijt*_	On day *t*, whether child *i* receive the *j*-th dose of the vaccine (0-1 variable: 1 if vaccinated, 0 otherwise).
*l*_*ijt*_	The earliest age at which the *i*-th child can receive the *j*-th vaccine on day *t*.
*D*_*ij*_(*t*)	On day *t*, the priority level of the *t*-th child relative to the *j*-th vaccine dose, where a higher value indicates higher priority.
$\bar{j}$	The vaccine with the highest priority, $\bar{j}\in J$, is the vaccine *j* for which *D*_*ij*_(*t*) takes the maximum value.
*D*_*i*_(*t*)	On day *t*, the composite priority of child *i*, *D*_*i*_(*t*) = *D*_*ij*_(*t*) = max(*D*_*ij*_(*t*)), *j* ∈ *J*, where a higher value indicates a higher priority.
*B*_*ijt*_	On day *t* of scheduling, whether child *i* missed the vaccination time for dose *j* (0-1 variable, 1 if missed, 0 otherwise).
*B*_*itotalT*_	Within the total scheduling time, whether the *i*-th child miss their vaccination appointment.
*W*	The maximum daily vaccination capacity determined by the resources available at the vaccination site.
$\bar{w}$	The set of restricted vaccination doses that ensures no child misses vaccination, $\bar{w}=\left({\bar{w}}_{1},{\bar{w}}_{2},{\bar{w}}_{3},...{\bar{w}}_{y}\right)$.
${\bar{w}}_{best}$	Optimal vaccination dose.
*w*_*yt*_	When selecting the restricted vaccination quota $\bar{w}={\bar{w}}_{y}$, the actual number of individuals scheduled for vaccination on day *t*.
*P*	Compliance rate.
*Y*_1_	Objective value representing the minimum feasible daily vaccination quota.
*Y*_2_	Maximum daily capacity reserved for flu vaccinations during the flu prevention period.
Decision variables	
*w*	A specific value for the vaccination quota.
*v*_*t*_	Service space reserved for influenza vaccinations on day *t*.

When scheduling reserved service slots for emergency influenza vaccinations during the flu prevention period, the scheduling cycle *T* is fixed at 1 day. Within this cycle, the goal is to maximize the number of reserved service slots. The inspection period is *CT* days, and the compliance rate is *P*. This means that the maximum reserved service capacity within the influenza prevention cycle must withstand the inspection that no Category I vaccines are missed in scheduling over the subsequent *CT* days. If it fails, the reserved service capacity must be reduced until the inspection passes. Since the flu prevention vaccination period is shorter than that for Category I vaccines, and administering flu prevention vaccines early in an outbreak helps curb its spread, the earlier and the larger the number of people vaccinated, the better the prevention effect. However, within a certain service capacity range, to avoid children missing vaccinations due to Category I vaccines giving up too much service space for influenza prevention vaccinations, it is necessary to test the service space given up each day to see if there are any missed Category I vaccinations. If the reserved capacity shows no missed vaccinations for children after a *CT*-day verification period, the reserved capacity is deemed feasible and can be allocated for influenza prevention vaccination.

### 2.2 Preprocessing step: determination of decision variables and related parameters

#### 2.2.1 Vaccination interval determination

For a scheduled vaccine designated as the *j*-th dose, its latest administration time depends solely on the vaccine itself, i.e., it is *O*_*j*_. However, its earliest vaccination age is constrained not only by the earliest possible time *L*_*j*_ for that specific dose but also by previously administered vaccines (including different doses of the same vaccine) and the minimum required interval between this vaccine and those others. Let the current time be *t*. Under the influence of vaccine *k* only, the vaccination age for vaccine *j* must not be earlier than *f*_*ikt*_ + *x*_*ikt*_ × *S*_*jk*_. Clearly, if vaccine *k* has not been administered, then *f*_*ikt*_ and *x*_*ikt*_ are both 0. And the value of this equation is 0, indicating that other unadministered vaccines do not affect the earliest vaccination age of vaccine *j*. If vaccine *k* has been administered, then *x*_*ikt*_ = 1, meaning the vaccination age of vaccine *j* must not be earlier than *f*_*ikt*_ + *S*_*jk*_. Therefore, under the influence of all other vaccines, the vaccination age for vaccine *j* must not be earlier than $\max_{k\in J}\left(f_{ikt}+x_{ikt}\times S_{jk}\right)$. Additionally, constrained by vaccine *j* itself, its vaccination age must not be earlier than *L*_*j*_. Therefore, for child *i* at time *t*, the earliest administration age for vaccine *j* is $l_{ijt}=\max\left(\max_{k\in j}\left(x_{ikt}\times S_{jk}+f_{ikt}\right),L_{j}\right)$.

In summary, the reasonable vaccination age range is


$$\begin{array}{l} \{\begin{array}{ll} \left[l_{ijt},O_{j}\right] & l_{ijt}\leq O_{j}\\ \emptyset & l_{ijt}>O_{j} \end{array} \end{array}$$

#### 2.2.2 Priority determination

To reasonably design the priority system, the following requirements apply: This priority system must clearly distinguish between the following three groups:

(a) Those meeting vaccination eligibility criteria (including those who missed the latest vaccination window and require catch-up doses);
(b) Those who have completed vaccination;
(c) Those who do not yet meet vaccination eligibility criteria.

Since group a represents the population to be included in scheduling plans, it is essential to ensure that the priority of group (a) is significantly higher than that of groups (b) and (c). It is preferable to distinguish them using symbols, with the former being positive and the latter being 0 or negative. This facilitates filtering data eligible for inclusion in scheduling plans. Additionally, since group (b) represents those already vaccinated, this category should be ignored during priority sorting. In this program, the priority expression for this group is set to *D*_*ij*_(*t*) = 0. To ensure fairness in priority assignment, the following expression is used for groups (a) and (c) (those not yet vaccinated):


$$\begin{array}{l} D_{ij}(t)=\{\begin{array}{ll} \left(a_{it}-O_{j}\right)+C\times \frac{a_{it}-l_{ijt}}{\left|a_{it}-l_{ijt}\right|} & a_{it}\neq l_{ijt}\\ \left(a_{it}-O_{j}\right)+C & a_{it}=l_{ijt} \end{array} \end{array}$$

In the priority function, *C* is introduced as a sufficiently large positive constant to ensure a clear distinction between children who are already eligible for vaccination and those who are not yet eligible. Specifically, *C* is used to make the sign of the priority score primarily determined by whether *a*_*it*_ − *O*_*j*_, so that eligible children are always ranked ahead of non-eligible children. In this study, *C* is chosen to satisfy $C>\max_{i,j,t}|a_{it}-O_{j}|$, so that when $\frac{a_{it}-l_{ijt}}{\left|a_{it}-l_{ijt}\right|}<0$, *D*_*ij*_ < 0; otherwise, $\frac{a_{it}-l_{ijt}}{\left|a_{it}-l_{ijt}\right|}>0$, *D*_*ij*_ > 0. In the empirical analysis, we use *C* = 1000. Then the child's overall priority, i.e., the highest priority among all vaccines, is *D*_*i*_(*t*) = max(*D*_*ij*_(*t*)), *j* ∈ *J*. Let vaccine *j* be the vaccine corresponding to the composite priority *D*_*i*_(*t*),then $D_{i}\left(t\right)=\max\left(D_{i\bar{j}}\left(t\right)\right)$.

#### 2.2.3 Determination of missed vaccination timing

The ultimate goal of the vaccination schedule is to achieve balanced daily vaccination volumes. However, in the process of finding the optimal schedule, all feasible solutions must ensure that no child misses the latest vaccination age for any vaccine. Considering the latest age at which a child might miss a vaccination, there are two possible scenarios: (1) Imminent miss: Child *i* has not received vaccine *j*, but the child's age has already exceeded the maximum vaccination age for that vaccine (*x*_*ijt*_ = 0, *a*_*it*_ > *O*_*j*_); (2) Already missed: Child *i* has received vaccine *j*, but at an age exceeding vaccine *j* latest administration age (*x*_*ijt*_ = 0, *f*_*ijt*_ > *O*_*j*_). Therefore, the discriminant defining whether the *j*-th vaccine for the *i*-th child on day *t* is missed is defined as follows:


$$\begin{array}{l} B_{ijt}=\{\begin{array}{ll} 1 & \left(x_{ijt}=0,a_{it}>O_{j})\text{ or }(x_{ijt}=1,f_{ijt}>O_{j}\right)\\ 0 & else \end{array} \end{array}$$

Where, 1 indicates that some children missed their vaccination schedule, and 0 indicates that no children missed their vaccination schedule.

If no specific vaccine is considered, the decision rule for whether the *i*-th child has missed any vaccination within the total scheduling time *total*_*T* is as follows:


$$\begin{array}{l} B_{itotal\text{\_}T}=\{\begin{array}{cc} 1 & \sum_{t}\sum_{j}B_{ijt}>0\\ 0 & else \end{array} \end{array}$$

#### 2.2.4 Determination of optimal vaccination threshold

Given that all vaccines are administered at the appropriate time, with the total vaccination period and total number of doses fixed, the optimal vaccination capacity for achieving the most balanced scheduling is the minimum value of $\bar{w}$ among all feasible solutions. This represents the maximum daily limit of individuals a vaccination site must serve while ensuring no vaccinations are missed. The determination process is as follows:

Assuming the current vaccination status is: *Q* doses remain to be scheduled, and a total of *q* days are required to complete the vaccinations, the average is *Q*/*q*. The currently available daily vaccination quotas are ${\bar{w}}_{1}$ and ${\bar{w}}_{2}$. When the fluctuation range of actual daily vaccinations is similar under these two vaccination volume constraints, and *w*_2*t*_ exceeds *w*_1*t*_ by *x* individuals, the daily vaccination volume allocations are respectively *w*_11_, *w*_12_, ⋯ , *w*_1*q*_ and *w*_21_, *w*_22_, ⋯ , *w*_2*q*_, satisfying condition $\max\left(w_{11},w_{12},\cdots \;,w_{1q}\right)\leq {\bar{w}}_{1}$, $\max\left(w_{21},w_{22},\cdots \;,w_{2q}\right)\leq {\bar{w}}_{2}$, *w*_1*t*_ = *w*_2*t*_ − *x*. Using variance to determine the balance between the two schedules, then


$$\begin{array}{l} S\left({\bar{w}}_{1}\right)=\frac{{\left(w_{11}-\frac{Q}{q}\right)}^{2}+\cdots +{\left(w_{1q}-\frac{Q}{q}\right)}^{2}}{q}\\ S\left({\bar{w}}_{2}\right)=\frac{{\left(w_{21}-\frac{Q}{q}\right)}^{2}+\cdots +{\left(w_{2q}-\frac{Q}{q}\right)}^{2}}{q} \end{array}$$

We have $S\left({\bar{w}}_{2}\right)-S\left({\bar{w}}_{1}\right)=x^{2}>0$. In comparison, ${\bar{w}}_{1}$ represents the optimal restricted vaccination dose. While ensuring no missed vaccinations for Category I vaccines, solutions become increasingly optimal as ${\bar{w}}_{1}$ approaches *Q*/*q*. However, in practical implementation, the total remaining vaccine doses remain uncertain due to variations in the total scheduling time selected, rendering the *Q*/*q* value unknown. Therefore, this value must be determined using the binary search method. Since the application of the binary search method relies on the monotonic feasibility property, if there are *n* candidate thresholds, the exhaustive method requires up to *n* feasibility checks in the worst case, whereas the binary search method requires at most *log*2*n* checks. For example, when *W* = 80 and integer thresholds within the interval [1, 80] are considered, the exhaustive method may require up to 80 feasibility checks, whereas the binary search method requires at most 7 checks. Therefore, the advantage of the binary search method lies in reducing the number of redundant scheduling feasibility evaluations. Specifically, during the solution process, the intermediate value within [0, *W*] is first selected as the constraint for the vaccination dose to perform preliminary scheduling. Based on the scheduling results, the discriminant *B*_*itotal*_*T*_ is used to determine whether this intermediate value constitutes a feasible solution, thereby assessing the viability of the scheduling plan. If the intermediate value is feasible, the next scheduling's constrained inoculation dose is selected from the intermediate values in the range [0, *W*/2]. If it is infeasible, the next scheduling's constrained inoculation dose is selected from the intermediate values in the range [*W*/2, *W*]. This process is repeated iteratively until the minimum constrained inoculation dose is found, which is the optimal constrained inoculation dose.

Therefore, when the daily restricted vaccination dose set is $\bar{w}=\left({\bar{w}}_{1},{\bar{w}}_{2},{\bar{w}}_{3},...{\bar{w}}_{y}\right)$, and ${\bar{w}}_{1}<{\bar{w}}_{2}<{\bar{w}}_{3},...<{\bar{w}}_{y}$, the optimal restricted vaccination dose that achieves the most balanced schedule is ${\bar{w}}_{1}$, i.e., ${\bar{w}}_{best}={\bar{w}}_{1}$.

### 2.3 Optimization model

#### 2.3.1 Balanced scheduling model for Category I vaccine administration

The objective of the balanced vaccination model for Category I vaccines is to optimize the vaccination schedule, minimize the daily vaccination volume, and optimize the model as shown in Equations 1–4. In the objective function, the vaccination schedule is more balanced when the model-generated minimized vaccination quantity is closer to the daily vaccine administration limit. In our examples, this proximity is quantified by the following equation: $p^{\prime }\left(t\right)=\min\left(p,{\bar{w}}_{y}\right)$, where ${\bar{w}}_{y}$ represents the daily vaccine allocation limit, *p* denotes the number of children requiring vaccination on day *t* according to the priority calculation rules, and *p*′(*t*) indicates the number of children added to the vaccination schedule on day *t*. Additionally, children included in the vaccination schedule may not fully comply with the program. In our case, we considered this through the following equation: $w\left(p^{\prime }\left(t\right),P\right)\to {\bar{w}}_{y}$, where *P* denotes the compliance rate per child. For example, when *P* = 1, the range is [0, 1]. The data for the child with the highest priority, *p*′(*t*) = 10, is selected and sorted by priority. The probability that each child receives the vaccination is 1, meaning that these 10 children will complete the vaccination according to the schedule; When *P* = 0.9, the range is [0, 0.9]. The data for the children with the highest priority (*p*′(*t*) = 10) is selected and sorted by priority. The probability that each child receives the vaccination is 0.9. Consequently, the final number of children who complete this vaccination is 9, but these may not necessarily be the top 9 children in terms of priority. Specifically, when the daily vaccination quota is ${\bar{w}}_{y}$ and the compliance rate is *P*, the actual number of vaccinated children *w* on day *t* is a function of ${\bar{w}}_{y}$ and *P*, and the number of children allocated for service approaches ${\bar{w}}_{y}$. Since the scheduling outcome is determined by the vaccination quota and compliance rate, a binary search is performed within the interval [0, *W*]. For each intermediate value *w*, *B*_*itotal*_*T*_(*w, P*) evaluates whether it satisfies the constraints, ultimately identifying the optimal vaccination quota. The objective function is defined as follows:


$$\begin{array}{ll} Y_{1} & =\min\left(w\right) \end{array}$$ (1)

$$\begin{array}{ll} s.t.\; & B_{itotal\text{\_}T}\left(w,P\right)=0 \end{array}$$ (2)

$$\begin{array}{l} \underset{k}{\sum }x_{ikt}\leq 1 \end{array}$$ (3)

$$\begin{array}{l} \underset{t}{\sum }x_{ikt}=1 \end{array}$$ (4)

Objective function (1) minimizes the restricted vaccination dose to reduce variance in the daily number of vaccinated individuals, thereby producing a more balanced scheduling outcome. Constraint (2) ensures that no child misses a vaccination throughout the scheduling period. Constraint (3) enforces the condition that a child may receive only one vaccine dose per day. Constraint (4) specifies that a single vaccine dose for a child cannot be administered over multiple days.

#### 2.3.2 Balanced vaccination scheduling model for flu outbreaks

During an influenza outbreak, the Centers for Disease Control and Prevention (CDC) typically mobilizes all available resources to implement comprehensive prevention and control measures. Therefore, during such periods, the maximum vaccination capacity *W* of the CDC is adopted as the combined maximum capacity for influenza vaccination and Category I vaccine administration during the outbreak. Let the space reserved for influenza on day *t* be denoted as *v*_*t*_(*v*_*t*_ ≤ *W*), the service capacity reserved for children's Category I vaccinations on that day is *W* − *v*_*t*_. Since influenza prevention vaccinations are highly effective in controlling outbreaks and earlier administration yields better results, a larger *v*_*t*_ during the influenza prevention period leads to better control outcomes. However, an excessively large *v*_*t*_ may cause some children to miss certain Category I vaccinations in subsequent scheduling due to reduced service capacity.

To test this scenario, consider a verification time point *CT*. For the subsequent period *CT* (*t* + 1, *t* + *CT*), perform pre-scheduling and simulate vaccinations based on the daily service capacity *W*. Since the initial conditions for pre-scheduling are determined by *v*_*t*_, *P*, and *W*, the corresponding discriminant is *B*_*i*(*t*+*CT*)_(*W, P, v*_*t*_). Considering *W* and *P* as known constants, it can be simplified to *B*_*i*(*t*+*CT*)_(*v*_*t*_). If no child misses, i.e., *B*_*i*(*t*+*CT*)_(*v*_*t*_) = 0, then *v*_*t*_ is valid. The optimal buffer size is the maximum value of *v*_*t*_ that satisfies *B*_*i*(*t*+*CT*)_(*v*_*t*_) = 0. In summary, the objective of the balanced vaccination scheduling for sudden influenza outbreaks is to maximize the reserve capacity while ensuring no missed vaccinations for Category I vaccines. The objective function is specifically expressed as follows:


$$\begin{array}{l} Y_{2}=\max\left(v_{t}\right)\;s.t.\;B_{i\left(t+T\right)}\left(v_{t}\right)=0 \end{array}$$ (5)

Additionally, to maximize the reservation space, this paper adopts the SEIAR influenza dynamics model proposed by Iuliano et al. (25) to depict the diffusion process of influenza viruses within populations. This model incorporates the incubation period characteristics of influenza and the ability of asymptomatic individuals to transmit the virus, thereby enabling precise and in-depth reflection of the transmission mechanisms of influenza viruses. The model population is divided into five categories: susceptible individuals (*S*), exposed individuals (*E*), infected individuals (*I*), asymptomatic carriers (*A*), and recovered individuals (*R*). According to the principles of infectious disease dynamics modeling, the differential equations for the SEIAR model without intervention, incorporating asymptomatic carriers, are expressed as follows:


$$\begin{array}{l} \frac{dS}{dt}=-\beta S\left(I+kA\right),\;\frac{dE}{dt}=\beta S\left(I+kA\right)-\left(1-\rho \right)\omega E-\rho \omega^{\prime }E\\ \frac{dI}{dt}=\left(1-\rho \right)\omega E-\gamma I,\;\frac{dA}{dt}=\rho \omega^{\prime }E-\gamma^{\prime }A\\ \frac{dR}{dt}=\gamma I+\gamma^{\prime }A \end{array}$$

Where β represents the disease transmission rate; *k* denotes the transmission coefficient of asymptomatic carriers; ω signifies the incubation period coefficient; ω′ indicates the incubation period coefficient for asymptomatic carriers; ρ represents the proportion of asymptomatic carriers; γ denotes the removal rate coefficient for symptomatic carriers; γ′ indicates the removal rate coefficient for asymptomatic carriers. Meanwhile, the differential equation is as follows:


$$\begin{array}{ll} S\left(t+\Delta t\right) & =S\left(t\right)-\beta S\left(I+kA\right)\Delta t\\ E\left(t+\Delta t\right) & =E\left(t\right)+\left(\beta S\left(I+kA\right)-\left(1-\rho \right)\omega E-\rho \omega^{\prime }E\right)\Delta t\\ I\left(t+\Delta t\right) & =I\left(t\right)+\left(\left(1-\rho \right)\omega E-\gamma I\right)\Delta t\\ A\left(t+\Delta t\right) & =A\left(t\right)+\left(\rho \omega^{\prime }E-\gamma^{\prime }A\right)\Delta t\\ R\left(t+\Delta t\right) & =R\left(t\right)+\left(\gamma I+\gamma^{\prime }A\right)\Delta t \end{array}$$

Furthermore, incorporating the vaccination status of the influenza reserve space into the SEIAR differential equation with Δ*t* = 1 and the optimal daily reserve space $v_{t}^{\prime }$ yields:


$$\begin{array}{ll} S\left(t+1\right) & =S\left(t\right)-\beta S\left(I+kA\right)-v_{t}^{\prime }\\ E\left(t+1\right) & =E\left(t\right)+\beta S\left(I+kA\right)-\left(1-\rho \right)\omega E-\rho \omega^{\prime }E\\ I\left(t+1\right) & =I\left(t\right)+\left(1-\rho \right)\omega E-\gamma I\\ A\left(t+1\right) & =A\left(t\right)+\rho \omega^{\prime }E-\gamma^{\prime }A\\ R\left(t+1\right) & =R\left(t\right)+\gamma I+\gamma^{\prime }A+v_{t}^{\prime } \end{array}$$

### 2.4 Statistical analysis

To evaluate the statistical significance of the model's performance, we employed the following tests. For the Category I vaccination scheduling comparison, Levene's test was used to assess equality of variances between the control group and the model-scheduled group, followed by Welch t-test for comparing mean daily vaccination volumes under unequal variances. For the influenza outbreak case study, since the SEIAR model produces deterministic outputs for fixed parameters, a parametric bootstrap approach (1,000 replications) was adopted to assess the robustness of peak infection reduction, with key epidemiological parameters (β, ρ, γ) perturbed uniformly within 10% of their baseline values. The significance of peak reduction was evaluated using a permutation test. Additionally, a Wilcoxon signed-rank test was performed to verify that the reserved capacity for influenza vaccination was significantly greater than zero. All statistical analyses were conducted in MATLAB, with significance set at α = 0.05.

## 3 Results

### 3.1 Case study on balanced scheduling for Category I vaccinations

This study uses vaccination data from children registered at the Shenzhen Nanshan District Center for Disease Control and Prevention (CDC) to empirically validate the proposed balanced scheduling model for Category I vaccines. The analysis proceeds through the following steps: First, raw data are preprocessed to remove missing or irrelevant information, followed by the creation of a baseline sample and a control sample. The baseline sample represents the initial vaccination status prior to scheduling, while the control sample serves for comparative analysis with the model's scheduling outcomes. Second, the balanced scheduling model for Category I vaccines is applied to the baseline sample to systematically schedule children's vaccinations. Finally, statistical analysis and effectiveness evaluation are performed on the scheduling results.

Data were obtained from the CDC's childhood vaccination records spanning January 1, 2016, to December 31, 2017. Specifically, the full-year data from January 1, 2016, to December 31, 2016, was selected as the baseline sample for scheduling. This baseline data was input into the balanced scheduling model for Category I vaccines, with January 1,2017, designated as Day 0 of the schedule. to pre-schedule vaccinations for the entire year of 2017 and generate corresponding actual vaccination outcomes. Concurrently, the original vaccination data from January 1,2017, to December 31,2017, served as the control group sample for comparative analysis against the model-scheduled actual vaccination data for the full year of 2017. Regarding sample size, the original base scheduling sample comprised 4,193 children receiving a total of 11,528 vaccine doses; the original control group sample included 4,812 children receiving a total of 13,971 vaccine doses. Specifically, during model solution, parameters were selected based on actual research findings: *P* = 0.9, *T* = 7, *W* = 80, *total*_*T* = 365.

**Figure 2:**
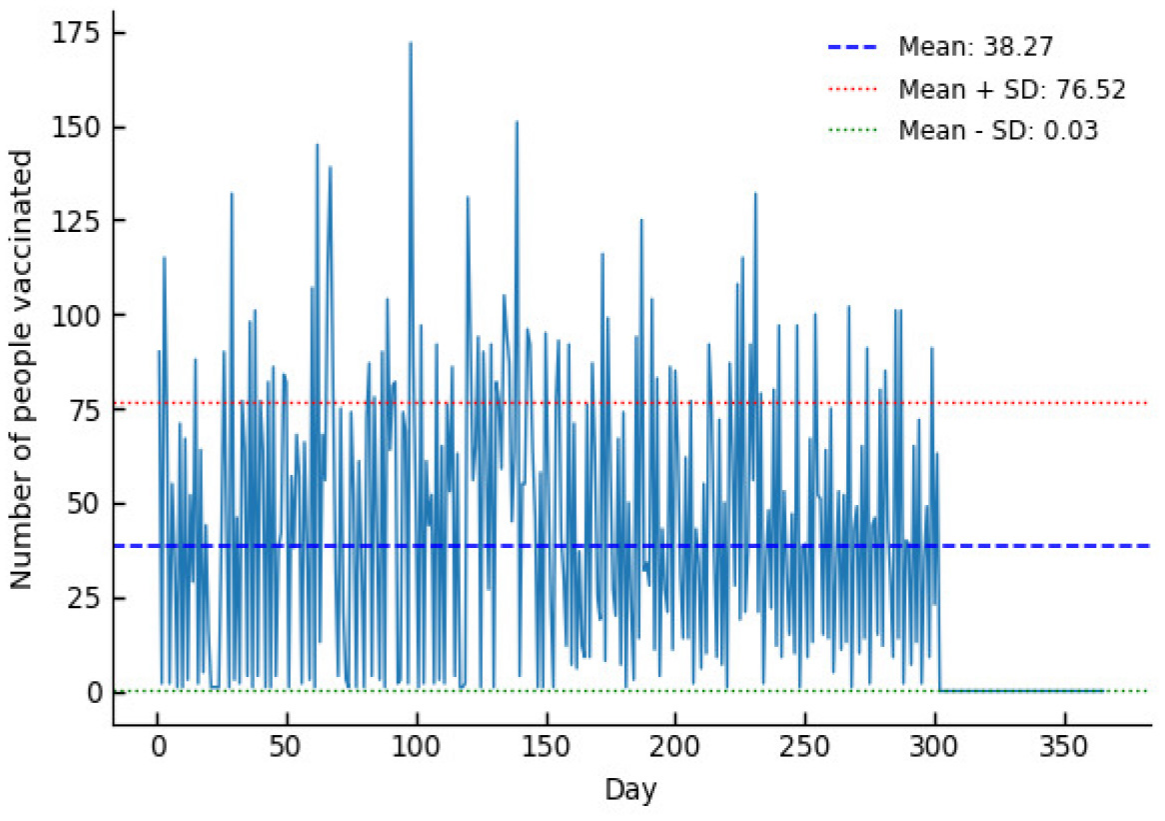
Distribution of vaccination status in the control group.

Data analysis was first conducted on the original control group sample to determine the daily vaccination distribution over the course of one year, as illustrated in Figure 2. The number of individuals vaccinated within the control group varied significantly across different periods of the year. Further analysis revealed an average of 38.27 vaccinated individuals per day, with a standard deviation of 38.25.

**Figure 3:**
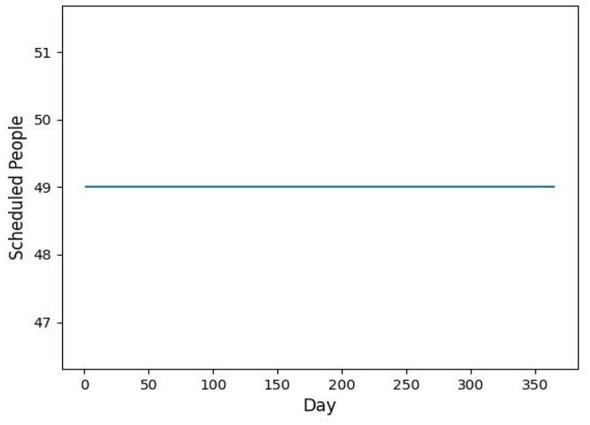
Number of people scheduled per day in the preliminary schedule.

**Figure 4:**
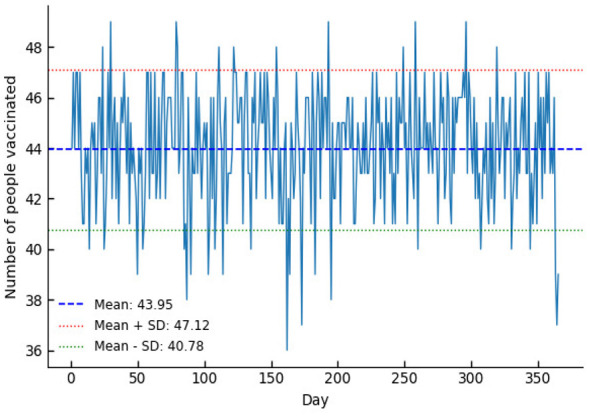
Annual vaccination schedule after scheduling.

The baseline sample was input into the Category I vaccination scheduling model. After running the simulation 10 times in MATLAB, the preliminary results yielded 1 instance of 43, 4 instances of 46, and 5 instances of 49. With a sufficiently large dataset, the results converged toward a specific value. In this case, the optimal restricted vaccination volume was determined to be 49 individuals, as shown in Figure 3. The preliminary results were then processed to exclude irrelevant data (e.g., cases where vaccination targets were not met), yielding the daily vaccination numbers for each center, as shown in Figure 4. The average daily vaccination count after scheduling was 43.95, with a standard deviation of 3.17, indicating a relatively balanced distribution of vaccinations throughout the year.

Comparing Figures 2, 4, the mean and standard deviation in the control group were 38 people and 38.25, respectively. After scheduling using the model proposed in this paper, the mean and variance of the scheduling results were 44 people and 3.17, respectively. The decrease in mean indicates that the model balanced resources over a longer time scale, while the reduction in standard deviation from 38.25 to 3.17 demonstrates that this model effectively improved the balance of the schedule.

To further validate the statistical significance of the scheduling improvement, we performed Levene's test for equality of variances and Welch's t-test on the daily vaccination counts. Levene's test yielded *F* = 412.87(*p* < 0.001), confirming that the variances of the two groups are significantly different. Welch's t-test further indicated a statistically significant difference in daily vaccination volumes between the control group and the model-scheduled group (*t* = −2.94, *p* = 0.004). These results proposed scheduling model significantly reduces daily vaccination variability while maintaining adequate coverage.

### 3.2 Case study on balanced scheduling for vaccination during a sudden influenza outbreak

This paper utilizes transmission data from Liang et al. (24) and Chen et al. (20) on an influenza outbreak a China secondary school as the scheduling baseline. The influenza parameters are as follows: β = 0.001, *k* = 0.5, ω = 0.5263, ω′ = 0.8333, ρ = 0.14, γ = 0.2128, γ′ = 0.2439. Specifically, during model solution, parameters were selected based on actual research findings: *P* = 0.9, *T* = 7, *DT* = 1, *W* = 80, *total*_*T* = 30, *CT* = 365. In particular, the most intensive vaccination period under China's NIP is concentrated between 0 and 730 days of age, and many vaccines have narrow vaccination windows (e.g., the first dose of polio vaccine at 30-60 days, and the first dose of DTP at 60–90 days). With a cycle time (CT) of 365 days, the system covers the full annual scheduling cycle and can account for all seasonal fluctuations in demand.

Based on the parameter settings outlined above, this study validated the theoretical model of balanced vaccination scheduling during an influenza outbreak through the following steps: (1) Relevant parameters were first substituted into the influenza transmission model without intervention to derive the disease's intrinsic propagation dynamics; (2) Next, the objective function for optimal service capacity reservation was used to determine the maximum daily service capacity allocated for influenza vaccination, ensuring that no Category I vaccinations were missed; (3) This result was then incorporated into the vaccination-adjusted influenza transmission model to derive the revised transmission dynamics; (4) By comparing transmission outcomes between the uninfluenced scenario and the vaccination-inclusive model, the effectiveness of influenza control was assessed, while simultaneously outputting the daily total number of vaccinated individuals and the daily reserved service capacity.

**Figure 5:**
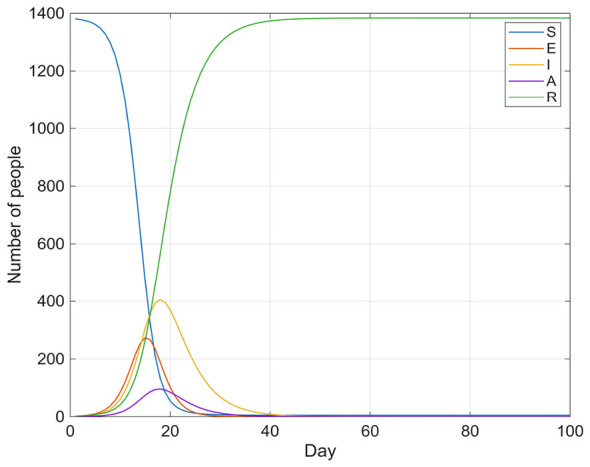
Influenza transmission without intervention.

**Figure 6:**
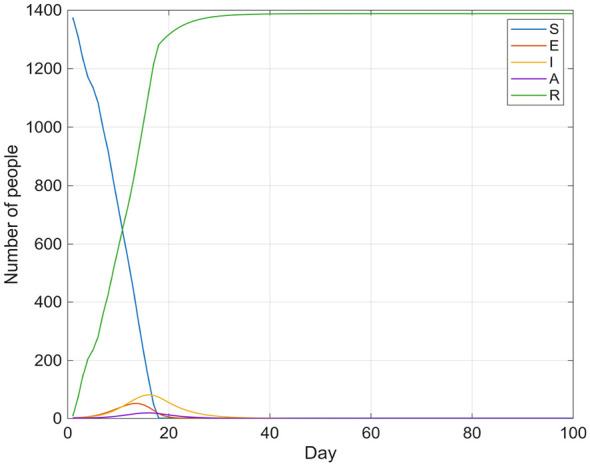
Influenza transmission (*W* = 80).

Figures 5, 6 show the trends in the number of infected individuals over time under no intervention and under consideration of emergency influenza vaccination, respectively.

Figures 5, 6 illustrate that both the number of symptomatic infections and the number of asymptomatic carriers increase gradually over approximately 10 days, peaking on days 15 and 18, respectively, before declining steadily. Subsequently, both values decrease monotonically to their minimums within about 30 days. It is evident that in the influenza transmission model without any intervention measures, the peak infection period (i.e., the sum of categories I and A) reached approximately 360 individuals, and the influenza outbreak was preliminarily controlled within about 30 days. Subsequently, the daily number of new infections remained at a low level of approximately 5 individuals. In contrast, when a flu vaccination schedule is introduced, the total peak infection count is significantly reduced to approximately 70 people, and the outbreak achieves complete control after day 33.

To assess the robustness and significance of the peak reduction, a parametric bootstrap analysis was conducted (1,000 replications). In each replication, the key epidemiological parameters (β, ρ, γ) were independently perturbed by sampling uniformly within 10% of their baseline values, and the SEIAR model was re-simulated under both the no-intervention and vaccination-intervention scenarios. The 95% bootstrap confidence interval for the absolute peak reduction (*I*+*A*) was [245, 335], and all 1,000 replications confirmed that the vaccination intervention yielded a lower peak than the no-intervention scenario (permutation test *p* < 0.001). These results indicate that the observed 80.6% reduction in peak infections is statistically robust across plausible parameter perturbations.

**Figure 7:**
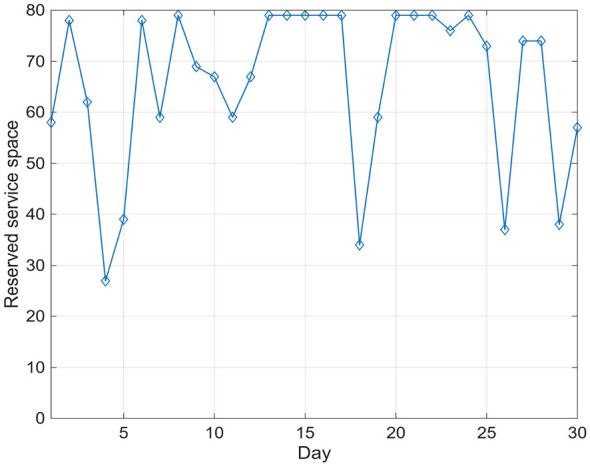
Service capacity reserved for influenza vaccines (*W* = 80).

Furthermore, when *W* = 80, Figure 7 and Table 2 present the daily number of Category I vaccine recipients and the optimal service capacity reserved for influenza vaccinations over 30 consecutive days, assuming a maximum daily capacity of 80 individuals at the vaccination site.

**Table 2:** Vaccination against influenza (*W* = 80).

Day	Total capacity	Category I vaccinated	Reserved capacity	Day	Total capacity	Category I vaccinated	Reserved capacity
1	80	22	58	16	80	1	79
2	80	2	78	17	80	1	79
3	80	18	62	18	80	46	34
4	80	53	27	19	80	21	59
5	80	41	39	20	80	1	79
6	80	2	78	21	80	1	79
7	80	21	59	22	80	1	79
8	80	1	79	23	80	4	76
9	80	11	69	24	80	1	79
10	80	13	67	25	80	7	73
11	80	21	59	26	80	43	37
12	80	13	67	27	80	6	74
13	80	1	79	28	80	6	74
14	80	1	79	29	80	42	38
15	80	1	79	30	80	23	57

As shown in Table 2, the daily reserved capacity for influenza vaccination exhibits significant fluctuations over time. This dynamic behavior is primarily driven by two factors: (1) Priority demand of Category I vaccinations. The number of children requiring high-priority vaccinations varies across days due to age eligibility windows and inter-dose interval constraints. When a larger number of children approach their latest allowable vaccination time, the system must allocate more capacity to Category I vaccines, thereby reducing the reserved capacity for influenza vaccination. For example, on Day 18, the number of Category I vaccinated individuals increases to 46, resulting in a reduced reserved capacity of 34; (2) Feasibility constraint under the verification horizon (CT days). The reserved capacity *v*_*t*_ must satisfy the constraint that no Category I vaccinations are missed in the subsequent CT days. When future scheduling pressure is high, the model restricts *v*_*t*_ to ensure feasibility. Conversely, when future demand is relatively low, more capacity can be allocated to influenza vaccination.This explains why on Days 16-17 and 20-22, where only 1 child requires Category I vaccination, the reserved capacity reaches as high as 79.

Furthermore, a one-sample Wilcoxon signed-rank test was performed on the 30 daily reserved capacity values (Table 2) to test whether the model reserves significant non-zero capacity for influenza vaccination. The median daily reserved capacity was 68.0 (*IQR*:38.75 − 79.0), and the test yielded *V* = 465 (*p* < 0.001), confirming that the scheduling model consistently allocates substantial service capacity for emergency influenza vaccination while ensuring no Category I vaccinations are missed.

Through the analysis of the above case, it can be observed that the balanced vaccination scheduling model can precisely calculate the daily service resources reserved for influenza vaccinations at vaccination sites. Compared to influenza transmission scenarios without intervention measures, balanced vaccination scheduling effectively reduces the number of infected individuals, fully validating its practicality in influenza prevention and control efforts. This approach enables the rational allocation of both influenza prevention resources and vaccination resources.

## 4 Conclusions

This paper proposes a novel and efficient optimization model for balanced scheduling strategies of routine vaccinations and emergency influenza vaccinations. Specifically, the model can (1) determine the minimum daily vaccination quota while ensuring no routine vaccinations are missed and (2) establish balanced schedules for vaccination centers based on children's ages and vaccination intervals. Furthermore, this balanced scheduling accounts for the actual compliance rate of children arriving for vaccination, preventing missed vaccinations among children who fail to attend scheduled appointments. (3) During influenza outbreaks, it balances scheduling for routine Category I vaccines and influenza vaccines at vaccination centers. This strategy ensures routine Category I vaccinations are not missed, reserves additional capacity for emergency influenza vaccinations, and maintains balanced daily vaccination volumes.

In this case study, we designed two scenarios to test the model's diverse applications and perspectives. Specifically, in a balanced scheduling analysis for a vaccine category, we selected two sets of childhood vaccination data from different fiscal years: one serving as the control group sample size and the other as the scheduling data baseline sample size. We then applied the balanced scheduling model to derive the actual vaccination data (control group sample for the current year). Comparing the actual scheduled vaccination data with the control group data revealed that the variance in the total number of vaccinated individuals throughout the year was significantly reduced after scheduling (control group variance: 38.25; scheduled variance: 3.17), validating the model's strong performance in achieving scheduling equilibrium. In the case study analyzing balanced scheduling for influenza vaccination during sudden outbreaks, comparisons were made between the number of influenza infections without intervention and the number after balanced scheduling. The number of infections decreased from 360 to 70 after scheduling, validating that the model can be effectively integrated with influenza prevention and control. It demonstrates that during influenza prevention periods, the model can still reasonably allocate both influenza vaccinations and Category I vaccinations. The case study demonstrates the generality and broad applicability of the model.

This model innovatively incorporates the real-world characteristic of children's vaccination compliance rates. Case studies demonstrate the importance of pre-scheduling when compliance rates exist. Specifically, at a given compliance rate, if a high-priority child fails to attend the vaccination station according to the scheduled plan, and no pre-scheduling is conducted in advance, that child will occupy a vaccination resource slot on that day. This results in the resource being wasted. Introducing pre-scheduling allows for subsequent actual vaccination scheduling based on the pre-scheduling plan, thereby eliminating wasted vaccination resources. This enhances the utilization rate of limited service resources. A limitation is that while the balanced vaccination scheduling model for sudden influenza outbreaks effectively integrates with influenza prevention and control-allowing reasonable allocation of both influenza prevention vaccinations and Category I vaccinations during outbreaks it does not prove that the total vaccination volume for these two categories represents the optimal vaccination volume. Furthermore, during case validation of the model, limitations in obtaining influenza data prevented testing the outbreak-considering model against different influenza outbreak scenarios, thus imposing certain limitations on the case results.

An additional limitation is the single-subtype assumption in the influenza transmission component. In practice, seasonal influenza may involve co-circulation of multiple subtypes with distinct transmission parameters and vaccine efficacy profiles. The single-subtype model may therefore underestimate total disease burden during multi-strain seasons, and the optimal reserved capacity *v*_*t*_ may require upward adjustment. Nevertheless, the core scheduling mechanism-dynamically allocating capacity between routine and emergency vaccination while ensuring no missed Category I doses remains structurally valid under multi-strain scenarios, as *v*_*t*_ can be recalculated using a multi-strain transmission model without modifying the scheduling framework itself. Extending the model to incorporate multi-strain dynamics represents a valuable direction for future work.

Future research could focus on the following three aspects: (1) In studies of balanced scheduling for Category I vaccinations, this paper did not account for individual vaccination preferences. Allowing children to receive vaccinations on any day within a designated time window, rather than on a specific day, could better address missed vaccinations and align more closely with practical realities; (2) In equilibrium scheduling models for influenza vaccination during outbreaks, adopting a global optimization approach could identify optimal allocation plans throughout the entire flu prevention period. This includes determining the total number of vaccinations and daily limits on the number of people scheduled for vaccination. By selectively reducing the daily allocation for influenza vaccination on certain days to prioritize high-priority Category I vaccinations, it may become possible to free up more vaccination capacity for influenza prevention in the future, thereby enhancing overall influenza control effectiveness; (3) Evaluating the model from multiple perspectives such as vaccination completion rates, timeliness, and utilization of vaccination services-will help provide a more comprehensive assessment of its effectiveness.

## Data Availability

The raw data supporting the conclusions of this article will be made available by the authors, without undue reservation.
