# Supplementary material for: Optimizing vaccination scheduling during influenza outbreaks: a SEIAR-based model for balancing routine and emergency vaccination demands
Source: Front Public Health. 2026 May 7;14:1807474. doi: 10.3389/fpubh.2026.1807474 (PMC13190471; doi:10.3389/fpubh.2026.1807474)
Supplement: Supplementary file 1 [file Supplementary_file_1.pdf]

Appendix

The Appendix provides a comprehensive schedule of vaccination intervals. Time windows for routine childhood Category I vaccinations are summarised in Table 1, while the minimum required inter-dose intervals for each vaccine are reported in Tables 2 and 3. Together, these tables specify vaccination requirements for doses 1-28 administered during childhood.

Table 1: Vaccination Time Windows for Numbered Vaccines (Unit: Days)

Vaccine Name	Dose 1	Dose 2	Dose 3	Dose 4
Hepatitis B Vaccine	0-1 (Dose 1)	1-30 (Dose 2)	90-360 (Dose 3)	-
BCG Vaccine	0-90 (Dose 4)	-	-	-
Polio Vaccine (Live/Inactivated)	30-60 (Dose 5)	60-90 (Dose 6)	90-120 (Dose 7)	120-1460 (Dose 8)
DPT Vaccine	60-90 (Dose 9)	90-120 (Dose 10)	120-150 (Dose 11)	150-547 (Dose 12)
Group A Meningococcal Polysaccharide Vaccine	150-182 (Dose 13)	272-547 (Dose 14)	-	-
Japanese Encephalitis Live Attenuated Vaccine	182-240 (Dose 15)	730-1095 (Dose 16)	-	-
Japanese Encephalitis Inactivated Vaccine	240-270 (Dose 17)	270-365 (Dose 18)	730-1095 (Dose 19)	2190-2555 (Dose 20)
Measles-Rubella Vaccine	240-365 (Dose 21)	-	-	-
Measles-Mumps-Rubella Vaccine	270-547 (Dose 22)	-	-	-
Hepatitis A Live Attenuated Vaccine	547-730 (Dose 23)	-	-	-
Hepatitis A Inactivated Vaccine	547-730 (Dose 24)	730-1095 (Dose 25)	-	-
Group A+C Meningococcal Polysaccharide Vaccine	1095-1460 (Dose 26)	2190-2555 (Dose 27)	-	-
DT Vaccine	2190-2555 (Dose 28)	-	-	-

Note: Data source: *National Immunization Program Guidelines (2016 Edition).*

Table 2: Vaccination Interval Requirements

Vaccine Type	Inter-dose Interval Requirements
Hepatitis B Vaccine	The interval between Dose 1 and Dose 2 must be 28 days. The interval between Dose 2 and Dose 3 must be 60 days.
BCG Vaccine	No requirements
Polio Vaccine (Live/Inactivated)	The interval between any two doses must be 28 days.
DPT Vaccine	The interval between any of the first three doses must be 28 days; the interval between Dose 3 and Dose 4 must be 180 days.
DT Vaccine	No requirements
Measles-Rubella Vaccine	No requirements
Measles-Mumps-Rubella Vaccine	No requirements
Japanese Encephalitis Live Attenuated Vaccine	No requirements
Japanese Encephalitis Inactivated Vaccine	The interval between the second and third doses must exceed 30 days and be less than one year, whereas the interval between the third and fourth doses must be at least three years.
Group A Meningococcal Polysaccharide Vaccine	The interval between Dose 1 and Dose 2 must be 90 days.
Group A+C Meningococcal Polysaccharide Vaccine	No requirements (Group A and Group A+C meningococcal polysaccharide vaccines are considered the same vaccine).
Hepatitis A Live Attenuated Vaccine	No requirements
Hepatitis A Inactivated Vaccine	The interval between doses must be 180 days.

Note: Data source: *National Immunization Program Guidelines (2016 Edition).*

Table 3: Specific Interval Schedule Between Vaccine Doses

$n \times n$	1	2	3	4	5	6	7	8	9	10	11	12	13	14	15	16	17	18	19	20	21	22	23	24	25	26	27	28
1	0	0	0	0	0	0	0	0	0	0	0	0	0	0	0	0	0	0	0	0	0	0	0	0	0	0	0	0
2	0	0	0	0	0	0	0	0	0	0	0	0	0	0	0	0	0	0	0	0	0	0	0	0	0	0	0	0
3	28	60	28	28	28	28	28	28	28	28	28	28	28	28	28	28	28	28	28	28	28	28	28	28	28	28	28	28
4	28	28	28	28	28	28	28	28	28	28	28	28	28	28	28	28	28	28	28	28	28	28	28	28	28	28	28	28
5	28	28	28	28	28	28	28	28	28	28	28	28	28	28	28	28	28	28	28	28	28	28	28	28	28	28	28	28
6	28	28	28	28	28	28	28	28	28	28	28	28	28	28	28	28	28	28	28	28	28	28	28	28	28	28	28	28
7	28	28	28	28	28	28	28	28	28	28	28	28	28	28	28	28	28	28	28	28	28	28	28	28	28	28	28	28
8	28	28	28	28	28	28	28	28	28	28	28	28	28	28	28	28	28	28	28	28	28	28	28	28	28	28	28	28
9	28	28	28	28	28	28	28	28	28	28	28	28	28	28	28	28	28	28	28	28	28	28	28	28	28	28	28	28
10	28	28	28	28	28	28	28	28	28	28	28	28	28	28	28	28	28	28	28	28	28	28	28	28	28	28	28	28
11	28	28	28	28	28	28	28	28	28	28	28	28	28	28	28	28	28	28	28	28	28	28	28	28	28	28	28	28
12	28	28	28	28	28	28	28	28	28	28	180	28	28	28	28	28	28	28	28	28	28	28	28	28	28	28	28	28
13	28	28	28	28	28	28	28	28	28	28	28	28	28	28	28	28	28	28	28	28	28	28	28	28	28	28	28	28
14	28	28	28	28	28	28	28	28	28	28	28	28	28	90	28	28	28	28	28	28	28	28	28	28	28	28	28	28
15	28	28	28	28	28	28	28	28	28	28	28	28	28	28	28	28	28	28	28	28	28	28	28	28	28	28	28	28
16	28	28	28	28	28	28	28	28	28	28	28	28	28	28	28	28	28	28	28	28	28	28	28	28	28	28	28	28
17	28	28	28	28	28	28	28	28	28	28	28	28	28	28	28	28	28	28	28	28	28	28	28	28	28	28	28	28
18	28	28	28	28	28	28	28	28	28	28	28	28	28	28	28	28	28	30	28	28	28	28	28	28	28	28	28	28
19	28	28	28	28	28	28	28	28	28	28	28	28	28	28	28	28	28	28	28	1095	28	28	28	28	28	28	28	28
20	28	28	28	28	28	28	28	28	28	28	28	28	28	28	28	28	28	28	28	28	28	28	28	28	28	28	28	28
21	28	28	28	28	28	28	28	28	28	28	28	28	28	28	28	28	28	28	28	28	28	28	28	28	28	28	28	28
22	28	28	28	28	28	28	28	28	28	28	28	28	28	28	28	28	28	28	28	28	28	28	28	28	28	28	28	28
23	28	28	28	28	28	28	28	28	28	28	28	28	28	28	28	28	28	28	28	28	28	28	28	28	28	28	28	28
24	28	28	28	28	28	28	28	28	28	28	28	28	28	28	28	28	28	28	28	28	28	28	28	28	180	28	28	28
25	28	28	28	28	28	28	28	28	28	28	28	28	28	28	28	28	28	28	28	28	28	28	28	28	28	28	28	28
26	28	28	28	28	28	28	28	28	28	28	28	28	28	28	28	28	28	28	28	28	28	28	28	28	28	28	28	28
27	28	28	28	28	28	28	28	28	28	28	28	28	28	28	28	28	28	28	28	28	28	28	28	28	28	28	28	28
28	28	28	28	28	28	28	28	28	28	28	28	28	28	28	28	28	28	28	28	28	28	28	28	28	28	28	28	28

Note: n represents the number of vaccine doses.
